# Some Unmodified Household Adsorbents for the Adsorption of Benzalkonium Chloride—A Kinetic and Thermodynamic Case Study for Commercially Available Paper

**DOI:** 10.3390/toxics11120950

**Published:** 2023-11-21

**Authors:** Enikő Bitay, Alexandra Csavdari

**Affiliations:** 1 Department of Mechanical Engineering, Faculty of Technical and Human Sciences, Sapientia Hungarian University of Transylvania, Calea Sighișoarei 2, 540485 Târgu-Mureş, Romania; 2 Bánki Donát Faculty of Mechanical and Safety Engineering, Óbuda University, Népszínház Street 8, 1081 Budapest, Hungary; 3 Research Institute of the Transylvanian Museum Society, Napoca Street 2-4, 400009 Cluj-Napoca, Romania; 4 Department of Chemical Engineering, Babeş-Bolyai University of Cluj-Napoca, Arany Janos Street 11, 400028 Cluj-Napoca, Romania; 5 Department of Analytical, Colloidal Chemistry and Technology of Rare Elements, Al-Farabi Kazakh National University, 71 Al-Farabi Avenue, Almaty 050040, Republic of Kazakhstan

**Keywords:** benzalkonium chloride, adsorption, unmodified adsorbents, household paper, pseudo-second-order kinetic law, chemisorption-dominated process

## Abstract

The extensive use of biocide surfactant benzalkonium chloride (BAC) during the SARS-CoV-2 pandemic has led to the buildup of this hazardous chemical in waste, surface and groundwater. e The study aims to elucidate whether various low-cost household materials are suitable, in their unmodified and untreated form, to effectively adsorb BAC from its aqueous solutions.. Additionally, if a proper adsorbent is identified, a description of the kinetics and thermodynamics of the process is also targeted. From among the five tested materials, a commercially available white household paper towel was chosen to best satisfy the criteria of low price, large availability, and standardization degree, as well as high adsorption capacity within a fairly short time window needed until equilibrium. Batch experiments were carried out with a commercial mixture of BAC-12 and BAC-14 within a temperature range of 18-45 degrees Celsius, and a 25-100 mg/g BAC/adsorbent initial mass ratio range, respectively. The overall process follows a pseudo-second-order kinetic law, with an apparent activation energy of 73.35 KJ/mole. Both the Langmuir and the Redlich–Peterson isotherms describe the equilibrium data at 298 K well, with a Gibbs free energy of −20.64 KJ/mole. These findings are in agreement with previous reports and suggest a hybrid but chemisorption-dominated process.

## 1. Introduction

**Scheme 1:**
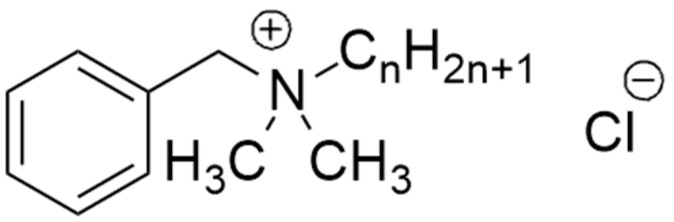
The structural formula of benzalkonium chloride (BAC), where n = 8 ÷ 18 stands for the even number of carbon atoms in the chain of the alkyl group.

Benzalkonium chloride (BAC) is a quaternary ammonium salt used mainly as a cationic surfactant with a broad anti-microbial spectrum. It may be observed from the structural formula illustrated in Scheme 1 that it contains a benzyl group, two methyl groups, and an alkyl group. The latter has a varying from 8 to 18, even-numbered carbon atom chain length. The commercially available formulation is usually a 50%wt aqueous BAC solution, containing a mixture of 12 and 14 carbon atom chains in different but usually equal ratios.

One of the drivers of its increasing market importance since the recent SARS-CoV-2-caused pandemic is the widespread use of BAC-containing hand sanitizers and surface disinfectants as alternatives to alcohol-based products. As a result, its market value reached USD 715.064 million in 2020 and is expected to grow by 8.7% by 2027 [1]. Although very effective for the purpose it has been designed for, BAC might accumulate in municipal or other wastewaters. Despite some efforts to inhibit its action or to biodegrade it [2,3], the usual corresponding wastewater treatment plants are not fully designed to deal with this species [4,5,6]. Hence, it may reach various natural environments and build up in wastewater influents and/or effluents, surface and groundwater, respectively [6], and as such, raise ecological concerns [7,8,9,10,11,12].

Although there is no official threshold limit for the legally allowed benzalkonium chloride content of surface, waste, and/or groundwater, chemical companies mention 96 h LC50 values for various fish in the range of ppm concentrations in the aquatic media [13,14]: 0.223 ÷ 0.46 mg/L for *Lepomis macrochirus*; 0.823 ÷ 1.61 mg/L for *Oncorhynchus mykiss*; 1.3 mg/L for *Poecilia reticulate*; and 2.4 mg/L for *Oryzias latipes*. Material safety sheets state that it is “very toxic to aquatic life” [14,15] and recommend that users avoid BAC discharge in lakes, streams, and sewers [15] and contact the environmental authorities in case of accidental leakages [15]. Fuchsman *et al.* [7] express the opinion that even though BAC-12 and BAC-14 (BAC species in Scheme 1, with n = 12 and 14) are the most prevalent BAC compounds in wastewater influents, they generally “exhibit low bioaccumulation potential, tending to be retained on epithelial surfaces rather than crossing cell membranes”. Hence, it is more likely for BAC-16 and BAC-18 (BAC species in Scheme 1, with n = 14 and 16) to be found in fish tissue [7].

It has been reported that BAC has a neurotoxic effect on zebrafish (*Danio rerio*). Sousa *et al.* [12] reported increasing overall swimming activity, thigmotaxis behavior, and erratic movements, whereas Sreevidya *et al.* [8] observed hatching delay or the inhibition of embryos, embryo enhanced mortality, and the prevalence of morphological malformations, with acute toxicity occurring at concentrations of hundreds of μg/L. Ikisa *et al*. [9] observed a direct positive relationship between *O. niloticus* juvenile mortality and BAC concentration in freshwater. BAC also induced relevant DNA damage, thus proving its genotoxicity, in both *Daphnia magna* and *Ceriodaphnia dubia* [10], with acute effects occurring at tens of μg/L in *D. magna* and hundreds of μg/L in *C. dubia*, respectively. On the other hand, comparable ranges of BAC concentrations of 15 to 200 μg/L did not significantly affect the physiological variables of *Isochrysis galbana* and *Chaetoceros gracilis* microalgae batch cultures, nor those of a coastal phytoplankton assemblage [11].

Tezel *et al.* [16] proved that at these low concentrations, counter-ions such as bromide, nitrate, and acetate did not affect the bioavailability, hence the toxicity of BAC, even if present in three-fold higher-order-of-magnitude concentrations. Yet, similar quantities of natural organic matter somewhat reduced the biocide’s toxicity. Reported environmentally relevant surface and freshwater BAC concentrations may vary from units of μg/L [10] to hundreds of μg/L [11,12], with levels inducing adverse effects or acute toxicity, depending on the examined species, anywhere within this range. Therefore, environmental risk issues created by the wide and intensive use of benzalkonium chloride may not be ignored when striving for the preservation of a safe and healthy environment [6].

Adsorption is one of the most versatile, efficient, and fairly low-cost methods to mitigate polluted waters from a wide variety of species [17,18], ranging from heavy metals [19] to cationic and/or anionic dyes [20,21]. Some studies have endeavored to use readily available low-cost adsorbents, such as sawdust [22,23,24], cardboard [24], or paper [25,26], whereas others have reported specifically designed ones in order to target certain adsorbates [27,28,29].

The removal of benzalkonium chloride from aqueous solutions by means of adsorption is also mentioned in the literature. One of the earliest studies reported that its adsorption on activated granular carbon [30] is controlled by intraparticle diffusion and characterized at equilibrium by a Freundlich isotherm. A decade ago, Zanini *et al.* [31] proposed the use of montmorillonite as an adsorbent and compared the adsorptions of BAC-12 and BAC-14, respectively. Both BAC variants exhibited two distinct stages in the isotherm, the one at lower pollutant concentrations corresponding almost exclusively to a cation exchange process, whereas the one at higher concentrations was considered to be mainly driven by lateral interactions between the surfactant BAC molecules. These studies were continued by adding triclosan, another biocide, to the pollutant’s mixture, as well as by putting forward a Langmuir isotherm for the binary adsorption model [32]. BAC’s competitive adsorption with a herbicide on the same adsorbent was assessed by means of a theoretical cation exchange model [33]. A laboratory-synthetized magnetite nanomaterial was also tried recently [34]. Again, a Langmuir isotherm described very well the experimental data.

Other types of BAC adsorbents include polymers. Both an acrylic ester, and a styrenic polymer were used to remove it from aqueous streams in a single adsorbent column [35]. Similar to previous findings [30], intraparticle mass transfer was considered to significantly affect the process rate. However, concerning the mechanism, the interaction between the adsorbent and adsorbate indicated slightly exothermic physical sorption [35]. The adsorption of three BAC variants (BAC-12, BAC-14, and BAC-16) on polyethylene micro-plastics, as well as their combined toxic effects on *Daphnia magna*, were assessed by using batch experiments [36]. Overall, pseudo-second-order kinetic laws were further proposed.

More detailed adsorption mechanisms involving various alkyl group chain length BACs were carried out just recently [37]. The adsorption of BAC-12, BAC-14 and BAC-16, respectively, from aqueous media on powdered activated carbon showed that species with longer alkyl chains were adsorbed more effectively. Therefore, electrostatic interaction is more significant in BAC-12 adsorption, whereas for the other two species, van der Waals interaction plays a more important role. In all cases, pseudo-second-order kinetics and Langmuir isotherms proved to best fit the experimental data.

BAC adsorption was employed for other purposes: to probe the montmorillonite surface reactivity when encapsulated in a composite of alginate hydrogels [38], to decide whether zeolite–surfactant–drug composites are suitable drug deliverers when BAC is co-adsorbed with sulfamethoxazole on purified natural clinoptilolite [39], or to choose the best sterilizing grade filter membranes in the pharmaceutical industry, so that they retain the least BAC possible, and its concentration in the filtrate does not drop below its predetermined specification sterile limit [40,41].

It may be concluded that although there was some preoccupation for benzalkonium chloride removal from various water samples by means of adsorption, research efforts have mostly concentrated on finding the process conditions (temperature, pH, mixing rate, adsorbate/adsorbent mass ratio, ionic strength, process time, etc.) that ensure the most effective BAC elimination. Furthermore, various process mechanisms and isotherm models have been proposed, whereas detailed kinetic aspects have rarely been addressed, almost entirely since the SARS-CoV-2 pandemic. Except for the filtering membranes, which were acquired form commercial sources, all other adsorbents mentioned in the literature were subjected to some physical or chemical treatment prior to the adsorption experiments.

Therefore, this work aims to test the suitability of low-cost, unmodified adsorbents for BAC among various household items. The proposed material ought to be employed effectively in is available form, either from commercial sources or waste. Additionally, supplementary experiments are planned concerning the suitable candidate in order to conclude upon a more detailed kinetic model of the overall process, as well as to put forward an isotherm equation that best explains the adsorption mechanism under the employed conditions.

## 2. Materials and Methods

A commercially available 50 wt% aqueous solution of benzalkonium chloride, containing a mixture of BAC-12 and BAC-14 in an unspecified ratio, was provided by *SC Grande Gloria SRL*, Galați, Romania and used as the source of a 5 g/L BAC stock solution. This was employed both in calibration (absorbance *vs.* BAC concentration) as well as in the adsorption experiments. All were carried out with deionized and double distilled water.

The following adsorbents were chosen from easily accessible sources: *Expertto* white household paper towel (*SC Grande Gloria SRL*, Galați, Romania), ”*Anna*” *Soft and Delicate* light blue viscose household cloth (*Stella Pack S.A.*, Lubartów, Poland), white tea filter paper (*Demmer Gmbh*, Wien, Austria), white cotton string (*Românofir S.A.*, Tălmaciu, Romania), and a mixture of oak and beech sawdust from a private mountain farm in Braşov County, Romania, respectively.

The adsorbent’s relative humidity was determined *via* gravimetric measurements (*Kern ABT220-5DNM* analytical scale) by drying samples of 20 g (*Memmert UN30* drying chamber) at 105 °C for 24 h and was assessed as a percentage of the mass before drying. The bulk density of dry sawdust was determined *via* the triplicate weighting of 200 mL solid. All adsorbents were employed in experiments in the form provided by their respective commercial sources, except the sawdust, which was dried first. No other physical or chemical treatment prior to the BAC adsorption experiments was employed.

Benzalkonium chloride adsorption experiments were carried out in batch arrangements by using 150 mL covered glass recipients in order to limit water evaporation during the course of the experiments. Each water–BAC–adsorbent mixture was prepared in triplicate in a 50 mL aqueous solution of 500 mg/L BAC. The start of the process was operationally defined by the moment at which a weighted amount of solid adsorbent was placed into the BAC solution. The variation of the initial adsorbate/adsorbent mass ratio in five steps, from 25 to 100 mg/g, was achieved by varying the mass of adsorbent placed into the recipients. The temperature was kept constant by pre-warming all of the employed materials and keeping the glasses in a thermostated drying chamber.

No stirring was employed during the experiments other than the rapid mixing of the sample when placing the adsorbent into it. The authors considered that such an approach might resemble the slow passing of wastewater through a filter.

At predetermined time intervals, varying from 30 min to 1–2 days, 3 mL aliquots of the liquid bulk solution were extracted in order to record their absorbance at 262 nm (*Jasco* V-650 UV-VIS spectrophotometer) by using quartz cuvettes of 1 cm optical path length. When 3 consecutive values did not vary for more than 2%, adsorption equilibrium was considered to be achieved. These aliquots were re-added to their corresponding mixtures in order to keep the liquid bulk’s volume constant during the experiment.

The remnant BAC concentration was calculated from the absorbance *vs*. BAC concentration Lambert-Beer calibration line. This was obtained by using 10 triplicate aqueous biocide solutions in the concentration range of 100 to 2500 mg/L BAC.

Necessary calculus has been carried out by using Microsoft Office Excel and its Data Analysis toolpak, as well as the MatLab Software (Version 2021 A).

## 3. Results and Discussion

### 3.1. Choice of Adsorbent

The five different adsorbent candidates were compared by means of the following criteria in order to choose the most suitable one for further studies:

1. Low price and easy availability (commercial sources);
2. Good standardization (already ensured by the producer);
3. An approximatelly zero value for the 262 nm absorbance of the adsorbent-water mixture, so that it does not interfere with the values provided by BAC;
4. A high equilibrium adsorption yield coupled with a low total S-L contact time required until equilibrium.

The first three criteria are summarized in Table 1. Prices were estimated by using the Romanian local currency conversion rate into EUR as of 30 October 2023. For the outer surface, only the apparent surface of both sides of a cloth or a sheet of paper is taken into account. It is not to be mistaken for the total surface available for adsorption, which also includes the inner surface of the pores. As such, the values in Table 1 were assessed *via* calculation from the geometrical size and weight measurements of one cloth, one sheet of household paper, and one tea filter, respectively. The parameter has no practical value except for being a helpful tool in assessing the standardization quality ensured by the producer. The same is true for the relative humidity. The listed absorbance at 262 nm represents the average value registered for five distinct mixtures within the range of 2.5–25 g/L solid adsorbent in water.

**Figure 1:**
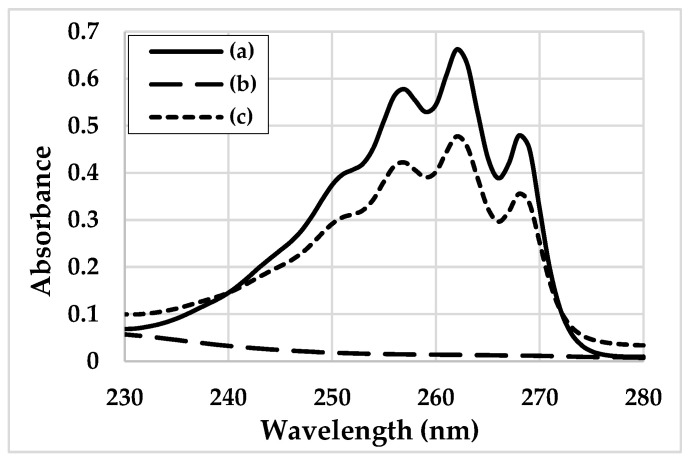
UV absorbance spectrum of the liquid phase for (a) a 500 mg/L aqueous BAC solution, (b) a water-paper mixture containing 10 g/L paper, and (c) a water-BAC-paper mixture containing initially 500 mg/L BAC and 10 g/L paper, at adsorption equilibrium and at 18.0 ± 0.1 °C, respectively.

Figure 1 sheds light on the choice of the use of a 262 nm wavelength to monitor the absorbance of the liquid phase in order to assess its remaining BAC content. Curve (a) corresponds to the UV spectrum of a 500 mg/L aqueous BAC solution. It has three peaks, at 268, 262 and 257 nm, respectively, with the one at 262 nm being the highest. Therefore, it was chosen for the purpose of quantifying BAC concentrations from the absorbance measurements as long as the adsorbent does not add to the A value. This is exactly the case with the white household paper towel. Its UV spectrum is illustrated in curve (b), and shows the negligible value of A = 0.0136 at 262 nm. Curve (c) illustrates the spectrum of the aqueous bulk phase of an initially 500 mg/L BAC solution containing 10 g/L paper at adsorption equilibrium (after 48 h of total S-L contact time). It may be observed that (i) the shape of the spectrum is similar to that of curve (a), demonstrating that the adsorbent does not affect A values, and (ii) the peak at 262 nm can be correlated with the remaining BAC content in the liquid phase. As a result, the difference of absorbance A at 262 nm between curves (a) and (c) is directly correlated with the adsorbed BAC. Methods relying on spectrophotometric determination of BAC use the same wavelength of 262 nm in UV [37] or a close one of 215 nm in the far-UV [41].

The data in Table 1 indicate sawdust as the first choice in terms of price. However, it presents the disadvantage of needing standardization as well as having a significant absorbance signal at 262 nm. This is due to some extraction–dissolution processes of organic species from sawdust [42,43]. Hence, it might interfere with the determination of BAC concentrations *via* spectrophotometric measurements and could potentially interfere with the BAC adsorption process. The next best option is the household paper towel. It has a reasonable price, the producer ensures a good standardization quality, and no other species interferes with BAC concentration determinations at 262 nm or with its adsorption.

**Figure 2:**
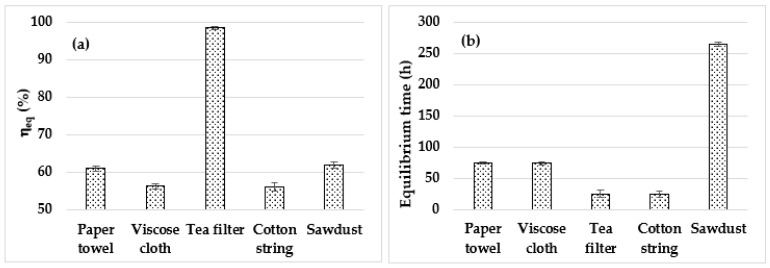
(**a**) Values of the equilibrium adsorption yield; (**b**) total S-L contact time until equilibrium. Data describe the 5 different employed adsorbents, with corresponding error bars for triplicate determinations, at 50 mg/g BAC/adsorbent initial mass ratio and 18.0 ± 0.1 °C, respectively.

Criterion 4 in the list above was assessed by using the equilibrium 262 nm absorbance values of the aqueous bulk solutions, A_eq_, of the water–BAC–adsorbent mixtures, as well as the corresponding A_0_ values of BAC aqueous solutions of the same concentration as the tests’ initial ones. In this study, 10 g/L of each adsorbent was employed for C^0^_BAC_ = 1000 mg/L initial BAC concentrations at 18.0 ± 0.1 °C. The adsorption equilibrium yield η_eq_ (%) was calculated by means of Equation (1). Equilibrium was considered to be achieved when three consecutive absorbance measurements (at different total S-L contact times) did not vary for more than 2%. Figure 2a,b illustrate the results registered during the assessment of criterion 4 for the choice of the most suitable BAC adsorbent.

(1) $$\eta_{eq}=\frac{A_{0}-A_{eq}}{A_{0}}\times 100$$

The calculus for η_eq_ was carried out with absorbance values instead of BAC concentrations because not all adsorbents have negligible A_262_ values in aqueous media, see sawdust, for example. The first four adsorbents in Table 1 do not add to the overall absorbance value at 262 nm, and the A values in Equation (1) are only due to the presence of BAC. Hence, the corresponding η_eq_ values of these adsorbents in Figure 2a are pertinent.

On the other hand, since the sawdust itself contributes to any A_262_ value registered for the bulk liquid phase, the A_eq_ value of the sawdust–BAC mixtures contains contributions from both species. As a result, the numerator (A_0_ − A_eq_) in Equation (1) is not quantitatively linked with the remaining liquid phase BAC concentration at equilibrium. Hence, the value of η_eq_ for sawdust in Figure 2a ought to be regarded with reservations.

According to the data presented in Figure 2, the most suitable adsorbent that satisfies criterion 4 above is tea filter paper. In 4 h, it reaches an approximately 80% adsorption yield, and in 24, almost 100%. The next suitable choices would be cotton string, household paper towel and household viscose cloth, in this order. Yet, the cheapest among these is the towel. According to the data in Table 1, it also would be a good choice.

Hence, the white household paper towel provided by *Expertto* (*SC Grande Gloria SRL*, Galați, Romania) was chosen for further BAC batch adsorption experiments for the purpose of establishing the kinetic and thermodynamic aspects of this process.

### 3.2. Experimental Data and Their Processing Method

The BAC adsorption experiments from its synthetic aqueous solutions on solid household paper towels were carried out, as described in Section 2. The one variable at a time approach was used. Firstly, the temperature varied stepwise at constant initial BAC and adsorbent contents in the mixtures (constant adsorbate/adsorbent initial mass ratio). Then, the temperature was kept constant at 25.0 ± 0.1 °C, and the adsorbate/adsorbent initial mass ratio varied stepwise. These data ought to elucidate the process thermodynamics and serve for the calculus of the equilibrium constant at 25 °C, the standard temperature.

Since, in all cases, the absorbance values were recorded at predetermined values of time, more often after the initiation of the process and less often towards the equilibrium, all data serve for the elucidation of process kinetics, as well as for the calculus of overall rate coefficients.

A calibration line absorbance *vs.* C_BAC_ was obtained at 262 nm in order to link the absorbance of the liquid phase bulk with the remaining, un-adsorbed BAC concentration. Its mathematical expression is provided in Equation (2), where C_BAC_ is expressed in mg/L.

A_262_ = (0.071 ± 0.014) + (1.184 ± 0.011)×10^−3^ C_BAC_ (2)

The R^2^_adjusted_ = 0.9974 value demonstrates the good quality of the calibration. The slope permits the calculus of the molar absorption coefficient for known BAC variants. Because BAC solutions have been prepared from a commercial source with an unspecified BAC-12 and BAC-14 ratio, concentrations are expressed in “overall” mg/L BAC instead of mole/L. Therefore, the slope of Equation (2) is expressed in (mg/L)^−1^·cm^−1^.

For correct C_BAC_ calculus, the A_262_ values used in Equation (2) were corrected by subtracting the adsorbent’s own absorbance (of an average value of 0.015 as listed in Table 1) from the one of the test’s mixture.

Further, C_BAC_ values served to calculate the adsorption capacity q (mg/g), according to Equation (3). C^0^_BAC_ (mg/L) stands for the initial concentration of BAC, whereas C_BAC_ (mg/L) stands for the one at a certain moment t after process initiation, respectively. C_adsorbent_ (g/L) is the adsorbent content of the aqueous BAC–solid adsorbent mixture. Hence, q stands for the mass (mg) of BAC adsorbed by 1 g of adsorbent after a certain S-L contact time t.

(3) $$q=\frac{C_{BAC}^{0}-C_{BAC}}{C_{adsorbent}}$$

The equilibrium value of q is represented by q_eq_ and was determined experimentally in each case.

**Figure 3:**
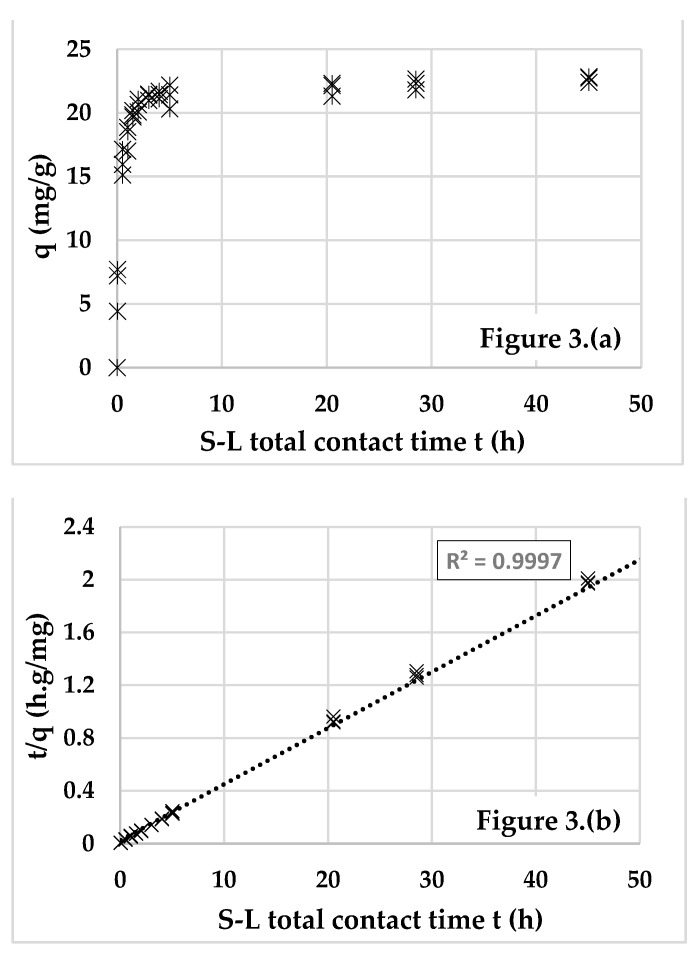
Example of adsorption capacity q *vs.* total S-L contact time plot (**a**), and the corresponding plot of the linearized pseudo-second-order kinetic law (**b**). Data for 50 mg/g initial BAC/paper mass ratio, at 25.0 ± 0.1 °C.

Figure 3a presents a set of typical results of calculus with Equation (3) in the form of the adsorption capacity q *vs.* total S-L contact time profile at 25.0 ± 0.1 °C and 50 mg/g initial BAC/household paper towel mass ratio. The star symbols stand for all of the data derived from the triplicate experiments. Negligible differences among the triplicates may be observed (RSD < 5%). The data show that approximately 90% of the process occurs within the first 5 h, with dramatic q value increases. Yet, equilibrium is attained far more slowly, taking about three or more days. These findings are in agreement with the data reported in the literature [31,36,37].

The first line of experiments was carried out at 18, 25, 35 and 45 °C, respectively, by keeping the starting BAC/paper mass ratio at constant 50 mg/g. The second line of trials kept the temperature constant at 25 °C, and varied the initial adsorbate/adsorbent mass ratio by giving it values of 25, 37.5, 50, 75 and 100 mg/g, respectively. In all cases, similar curves to the one depicted in Figure 3a were obtained, with significantly shorter time frames necessary until equilibrium at higher temperatures.

Single-factor ANOVA statistical testing was employed in order to assess whether the *q = f(time)* profiles like the one in Figure 3a were statistically significantly affected by the variation of either the temperature or the initial adsorbate/adsorbent mass ratio. Calculus was performed by using the Data Analysis toolpak of Microsoft Office Excel.

Statistical testing based on analysis of variance (ANOVA) is a technique that allows the isolation and estimation of variations between various data sets. It further classifies the differences as statistically “significant” or “insignificant”, depending on the value of the Fisher criterion (the F value). If its value exceeds the F_significance_ threshold, then the differences are statistically significant [44] (pp. 59–65). Otherwise, they are statistically insignificant. The threshold value depends on the number of degrees of freedom, which, in turn, is related to the number of experimental data subjected to testing.

For the effect of temperature, the value of the Fisher criterion was found to be equal to 17.62, higher than the threshold F_significance_ = 2.68. Similar results were obtained for the effect of the initial BAC/paper mass ratio, with F = 1874.4 being much higher than F_significance_ = 2.41. The results show that both parameters affect statistically significantly the course and outcome of the adsorption process, yet with a far more dramatic influence by initial conditions than the temperature (at least within the range of employed process conditions).

### 3.3. Process Kinetics

The *q = f(time)* profiles obtained *via* the above-described means have been tested against the pseudo-first, pseudo-second, and intra-particle diffusion models described by Yagub *et al.* [21], as well as against a more detailed kinetic model consisting of two parallel competing first-order individual stages [45]. The only fit was the one describing a pseudo-second-order overall adsorption process. According to it, the value of q follows a hyperbolic profile until it reaches the equilibrium value q_eq_, see Figure 3a, with a pseudo-second-order kinetic rate coefficient k expressed in (g/mg·h), see Equation (4).

(4) $$\frac{dq}{dt}=k{\left(q_{eq}-q\right)}^{2}$$

Its linear form (5) permits the calculus of both the rate coefficient ***k*** and the equilibrium adsorption capacity q_eq_, from the intercept and slope of the linear plot of the (t/q) values against t. Figure 3b presents this for the data shown in Figure 3a. Good linearization is observed, proving the suitability of the chosen kinetic model.

(5) $$\frac{t}{q}=\frac{1}{kq_{eq}^{2}}+\frac{1}{q_{eq}}t$$

The plot in Figure 3b enabled the calculation of q_eq_ = 23.53 mg/g, which is practically equal to the experimental 23.56 mg/g value (see also Table 3). Similar matches were found for the other employed experimental conditions. This fact is a further argument in favor of the kinetic model described in Equation (4). Such findings are in agreement with previously reported ones [36,37].

**Figure 4:**
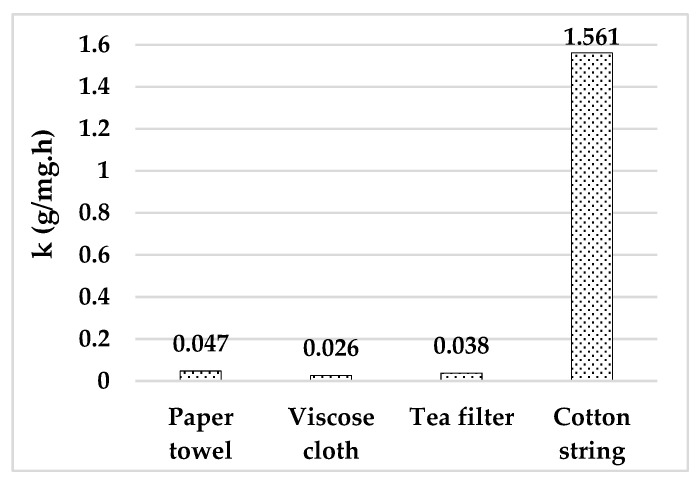
Pseudo-second-order rate coefficients k for various adsorbents at 18.0 ± 0.1 °C and 50 mg/g initial BAC/adsorbent mass ratio.

Equation (5) was used to determine adsorption rate coefficients for three more adsorbents of Table 1: household viscose cloth, tea filter paper, and cotton string, respectively, at 18.0 ± 0.1 °C and 50 mg/g adsorbate/adsorbent initial mass ratio. For these species, the calculus algorithm, described in Section 3.2, was also suitable because the adsorbent did not contribute to the overall absorbance value of the bulk liquid phase at 262 nm. The results are summarized in Figure 4 and are in good correlation with those presented in Figure 2b, supporting the decision favoring the use of this paper towel as a suitable BAC absorbent.

Table 2 contains the calculated pseudo-second-order rate coefficients k as a function of the employed temperature, T, as well as the statistical R^2^_adjusted_ values of the linear regressions from which they were calculated, such as the one in Figure 3b. The last column proves the good quality of the (i) experimental data and (ii) linearizations, hence model fit.

R^2^_adjusted_ is a strong statistical tool when comparing models with different numbers of independent variables *m* and data points *n*. Therefore, it is a better indicator of model quality than the simple R^2^, because it shows the variability in the target variable due only to independent variables that actually affect it [44] (pp. 92–108), [46,47]. The relationship between these two statistical parameters is described in Equation (6). It may be observed that, for the same *m* = 1 (the case here), if *n* differs, so will R^2^, and hence R^2^_adjusted_. The value of the latter being close to 1 demonstrates that the data fit very well with the proposed equation, in this case, with the second-order kinetic law.

(6) $$R_{adjusted}^{2}=1-\left(1-R^{2}\right)\left[\frac{n-1}{n-\left(m-1\right)}\right]$$

The rate coefficient values in Table 2 have been used to calculate the overall activation energy E_a_ and the pre-exponential factor Z by means of the linearized Arrhenius law. It fits fairly well (R^2^_adjusted_ = 0.7855) with the four experimental ln(k) *vs*. 1/T values. Hence, E_a_ = 73.35 ± 21.26 KJ/mole and ln(Z) = 27.01 ± 8.43 were obtained, pointing to the chemisorption of BAC as the main adsorption mechanism. Previously, similar [31] and opposite (physical sorption) findings [35] have been previously reported, mainly depending on the nature of the adsorbent but also on the initial adsorbate/adsorbent mass ratio.

**Figure 5:**
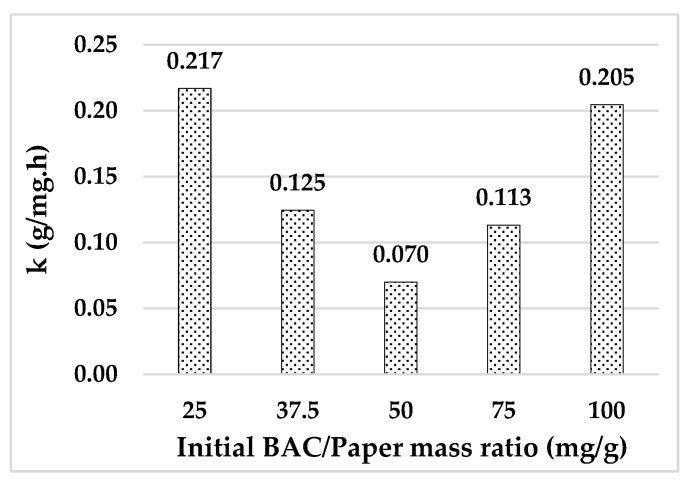
The dependence of overall pseudo-second-order rate coefficients k on the BAC/paper initial mass ratio, at 25.0 ± 0.1 °C.

The experiments carried out at a constant 25 °C and variable initial BAC/paper mass ratio generated the rate coefficient values presented in Figure 5. It shows that the overall adsorption process is favored by both low and high ratios. At low ones, the adsorption might be favored by the large number of available empty sorption sites, thus occurring faster. On the other hand, at high mass ratios, the concentration gradient of BAC between the liquid bulk phase and the adsorbent’s surface increases, favoring diffusion, hence raising the overall rate constant.

### 3.4. Adsorption Isotherms and Mechanism

The experiments carried out until equilibrium under the conditions described in Figure 5 led to the data presented in Table 3. It contains the equilibrium adsorption capacities q_eq_ (mg/g), which are quantitatively linked to the BAC retained on the adsorbent’s surface as a function of their corresponding equilibrium remnant concentration in the liquid bulk phase, C_eq_ (mg/L). Both experimental and calculated q_eq_ values are provided. The latter are obtained from plots described through the use of Equation (5) and exemplified by Figure 3b. There is a very good match between the q_eq_ value pairs. Hence, (i) the suitability of the second-order kinetic model is reinforced since the slope of its linearized form (5) serves for the determination of q_eq_ calculated and (ii) the good quality of the experimental data is proven (see also the corresponding excellent R^2^_adjusted_ values).

Despite the fact that only the Langmuir [32,34,37] and Freundlich [30] isotherms are mentioned in the literature, the fitting quality of eight different adsorption isotherms [47,48,49,50] over these data was evaluated in order to decide upon one that might elucidate the process mechanism. Four models have two parameters (Langmuir, Freundlich, Dubinin-Radushkevich and Temkin), and four have three (Hill, Redlich–Peterson, Toth, and Radke–Prausnitz), respectively (see also Table 4).

The Langmuir model describes the ideal situation for which all adsorption sites are homogeneous in structure and, therefore, energetically equivalent. The adsorption is assumed to take place in a monolayer, one site at a time, only through chemisorption, without any consequences on the neighboring positions of an occupied site. Its equation is presented in Table 4. Its linearized form in Equation (7) permits the easy calculus of both the maximum adsorption capacity of a single layer at a given temperature, q_max_ (mg/g), and the adsorption equilibrium constant at that temperature, K_L_ (L/mg).

(7) $$\frac{C_{eq}}{q_{eq}}=\frac{C_{eq}}{q_{max}}+\frac{1}{K_{L}q_{max}}$$

The data presented in Table 3 fitted well Equation (7), with R^2^_adjsuted_ = 0.9562. Hence, at the standard temperature of 298 K, the following were obtained: q_max_ = 35.09 mg/g and K_L_ = 11.76 L/g, respectively.

However, despite being easy, the use of simple linear regressions to evaluate the fitting quality and to calculate the parameters of a model can generate significant errors. Moreover, it is limited to models that allow linearization. Therefore, the parameters of the eight isotherms were calculated by means of an optimization algorithm in MatLab Software. This works with the normalized mean square error (NMSE) as an objective function and strives to find its minimum. Its expression is provided in Equation (8). q_exp(i)_ and q_calc(i)_ stand for the i^th^ experimental and calculated q values, respectively. The denominator represents the average value of q_exp_, and n is the number of experiments (here n = 5). The decision variables of the objective function are the sought-after model parameters.

(8) $$NMSE=\frac{\sqrt{\frac{\sum_{i=1}^{n}{\left(q_{exp(i)}-q_{calc(i)}\right)}^{2}}{n}}}{\bar{q_{exp}}}$$

After finding the parameters of each adsorption isotherm, three more error functions were calculated as simultaneous tools to be used when deciding upon the best model: (1) RMSD function (root mean squared deviations), see Equation (9), (2) HYBRID function (hybrid fractional error deviation), see Equation (10) in which p is the number of parameters of the model [42,50], and (3) R^2^_adjusted_, see Equation (6).

(9) $$RMSD=\sqrt{\frac{\sum_{i=1}^{n}{\left(q_{exp(i)}-q_{calc(i)}\right)}^{2}}{n}}$$

(10) $$HYBRID=\frac{100}{n-p}\sum_{i=1}^{n}{\left(\frac{q_{exp(i)}-q_{calc(i)}}{q_{exp(i)}}\right)}^{2}$$

Table 4 lists the calculus results for the eight isotherms taken into consideration: the value of their parameters as well as of the three above-mentioned error functions. The footnotes indicate the units of the parameters for each isotherm [49,50]. A good quality fit simultaneously satisfies the following requests: R^2^_adjusted_ as close to 1 as possible, whereas RMSD and HYBRID ought to be as close to zero as possible.

**Figure 6:**
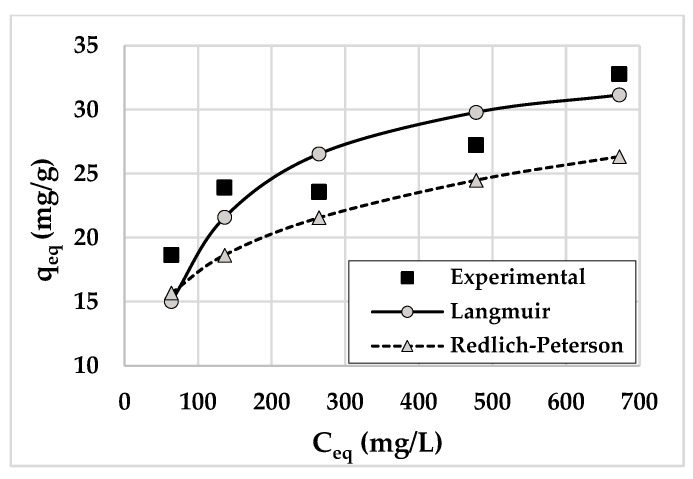
Experimental equilibrium isotherm data *vs.* calculated data for the Langmuir and Redlich–Peterson isotherms, respectively, at 25.0 ± 0.1 °C.

The analysis of the values in Table 4 suggests that the models fitting the best data in Table 3 are the Redlich–Peterson (RP) isotherm (lowest RMSD, relatively low HYBRID, very good R^2^_adjusted_ of 0.988), seconded by the Langmuir (L) isotherm (best R^2^_adjusted_, yet weakest HYBRID), respectively. A comparison between the experimental values of q_eq_ and those calculated by these two models is presented in Figure 6. Both seem to do a good job (see also results of Table 4); however, the L-isotherm predicts values that are scattered among the experimental ones, whereas the RP-equation predicts lower q_eq_ values, regardless of the C_eq_ level. Hence, despite the better statistical error functions of the RP model, the Langmuir one still holds best. Similar conclusions have been previously reported [32,34,37].

The RP isotherm combines the characteristics of both the Langmuir and Freundlich (F) ones [48,49]. Unlike the L-model, it does not assume ideal monolayer adsorption. The equation in Table 4 shows that when the parameter *g* = 1, it will reduce to the Langmuir relationship. On the other hand, at high liquid-phase adsorbate concentrations or low *g* values, the model reduces to the F-isotherm. In this case, the value of *g* = 0.780 shows a stronger similarity with the L-model. This fact also supports the conclusions reached in Figure 6.

Kim *et al.* [37] mention somewhat different sorption mechanisms for various BAC molecules: electrostatic interaction seems to be more significant in the case of BAC-12, whereas van der Waals bonding is more relevant for BAC molecules with longer alkyl chains. Since this study was carried out with a mixture of BAC-12 and BAC-14 in an unknown ratio, the equilibrium data describe a rather overall process, which seems to mainly follow chemisorption, with some physisorption (condensation) going on at high BAC concentrations: after completion of the monolayer, further BAC molecules can still attach themselves to the adsorbent’s surface *via* Van der Waals bonding to the previous layers.

These conclusions are supported by the findings of Zanini *et al.* [31], who studied the synergistic effect of BAC-12 and BAC-14 when simultaneously adsorbed from mixtures. An isotherm with two well-differentiated steps was proposed, each corresponding to a different adsorption mechanism. These impregnate a letter S-shaped q_eq_ *vs.* C_eq_ plot, each plateau (leveling off) corresponding to a step: the first being explained by chemical and the second by physical processes. The experimental data in Figure 6 exhibit such a shape somewhat, with a leveling off in the 100–320 mg/L C_eq_ domain. However, there are too few points to correctly apply a double L-model (a sum of two terms, like the Langmuir equation presented in Table 4) [31], with four parameters to be determined from only five q_eq_–C_eq_ pairs.

It is possible to estimate the Gibbs free energy of adsorption ΔG^0^ by using the Van’t Hoff relationship (11) and the value of the equilibrium constant; however, the latter ought to be dimensionless [51].

(11) $$∆G^{0}=-RT\ln K_{e}^{0}$$

Yet, the K_L_ = 11.76 L/g provided by the Langmuir model is not dimensionless. Equation (12) [51] describes a way of computing the dimensionless equilibrium constant K^0^_e_ in such cases:

(12) $$K_{e}^{0}=K_{L}\cdot \frac{\left[adsorbate\right]/{\left[adsorbate\right]}^{0}}{\gamma }$$

The [adsorbate]/[adsorbate]^0^ = 353.5 g/mole is the dimensionless standardized average molar mass of the employed BAC-12 and BAC-14 mixture (the ratio is unspecified by the provider; however, for the sake of this calculus, a 1:1 value was considered); oγ is the activity coefficient, which was here considered to be equal to 1. The obtained K^0^_e_ value enabled the calculus of ΔG^0^ with the help of (11) (R *=* 8.314 J/mole·K, T *=* 298 K).

The resulting value of the ΔG^0^ = −20.64 KJ/mole indicates a spontaneous process at 298 K. The magnitude of this adsorption free energy is comparable to those reported for other cationic adsorbates [52], yet it is approximately five-fold higher than values reported for BAC physisorption on powdered activated carbon [37]. Hence, again, a somewhat hybrid chemi- and physisorption mechanism may be assumed for the adsorption of BAC on the studied household paper.

This study proposes a low-cost, unmodified adsorbent for benzalkonium chloride from its aqueous solutions prepared by using a commercial mixture of BAC-12 and BAC-14. Both are readily accessible to the general public, both in terms of availability as well as price. Even so, the achieved adsorption capacities at 25 °C are comparable to the ones mentioned previously for another low-cost adsorbent: 11 ÷ 22 mg/g on polyethylene microplastics [40]. On the other hand, up to one order of magnitude higher values of (100 to 500 mg/g) may be targeted when using activated carbon [30,37] or montmorillonite [31,33,36,38]. However, these require complex and pricey physical and chemical treatment prior to their employment as adsorbents.

## 4. Conclusions

This study had the following aims: (1) to elucidate whether various low-cost household materials are suitable, in their unmodified and untreated form, to effectively adsorb benzalkonium chloride from its aqueous solutions and (2) to elucidate the process kinetics and thermodynamics of BAC adsorption on the proper adsorbent.

Among the five investigated adsorbents, household paper was chosen to be the most suitable for further testing in standardized batch experiments. Variation of both temperature and initial adsorbate/adsorbent mass ratio affected the adsorption capacity *vs.* the S-L contact time profile, with the latter being the stronger factor.

Four different kinetic models were tested, yet only the pseudo-second-order law fitted the data. Calculated rate coefficients lead to an apparent activation energy, which suggests that the removal of BAC occurs by means of chemisorption. Low and high initial BAC amounts in the sample speed up the process, which is either due to available adsorbent empty sites or the higher concentration gradient between the solid and liquid phases, respectively.

Equilibrium data at 25 °C were tested against eight isotherms. Their parameters were determined by minimizing the normalized mean square error. The data fitting quality was assessed through the use of three simultaneously employed statistical error functions. Both the Langmuir and the Redlich–Peterson isotherm were good fits, with the former being somewhat better scattered among the experimental values. The negative and medium-sized value of the Gibbs free energy suggested hybrid chemical and physical adsorption.

The study proved that even readily available, unmodified, and untreated household materials can be used as efficient adsorbents for the rapid removal of benzalkonium chloride, a biocide with many use yet which poses increasing ecological concerns.

## Figures and Tables

**Table 1:** Comparison among solid BAC adsorbent candidates in terms of price, availability, standardization degree and 262 nm absorbance value in water.

Adsorbent	Price (EUR/kg)	Availability	Standardization Degree ^1^	Absorbance at 262 nm
White household paper towel	7.5	Easy (retail)	− Controlled and constant quality ensured by the producer; − (42.68 ± 0.18) g/m^2^ outer surface; − (6.42 ± 0.24)% relative humidity.	0.015 ± 0.012
Light blue viscose household cloth	30.2	Easy (retail)	− 70% viscose, 30% polyester ^2^; − Controlled and constant quality ensured by the producer; − Approximately 18.9 g/m^2^ outer surface.	0.018 ± 0.009
White tea filter paper	82.2	Easy (retail)	− Controlled and constant quality ensured by the producer; − (16.99 ± 0.05) g/m^2^ outer surface; − (4.31 ± 0.17)% relative humidity.	0.018 ± 0.008
White cotton string	16.7	Easy (retail)	− Controlled and constant quality ensured by the producer; − Thickness^2^ of 1 mm; − Approximately^2^ 250 g/100 m.	0.003 ± 0.003
Sawdust	0.16	Easy (household)	− Quality and composition dependent on source; − Approximately 24% relative humidity; − (254.5 ± 3.8) g/L dry bulk density.	From 0.38 to 3.60

^1^ As ensured by the producer. ^2^ As indicated by the producer.

**Table 2:** Pseudo-second-order rate coefficients k as a function of temperature, at 50 mg/g initial BAC/household paper towel mass ratio.

T (K)	100 × k (g/mg·h)	R^2^_adjusted_
291 ± 0.1	4.74 ± 0.08	0.9932
298 ± 0.1	7.00 ± 0.23	0.9997
308 ± 0.1	10.44 ± 0.17	0.9971
318 ± 0.1	72.93 ± 1.68	0.9889

**Table 3:** Equilibrium data: BAC adsorption capacities q_eq_ (mg/g) *vs*. their corresponding equilibrium remnant concentration in the liquid bulk phase, C_eq_ (mg/L). Experimental and calculated data, at 25 ± 0.1 °C.

C_eq_ (mg/L)	q_eq_—Experimental (mg/g)	q_eq_—Calculated (mg/g)	R^2^_adjusted_
63.63 ± 4.03	18.64 ± 0.02	18.69 ± 0.04	0.9980
135.98 ± 2.09	23.90 ± 0.07	23.87 ± 0.02	0.9998
264.36 ± 2.11	23.56 ± 1.04	23.53 ± 1.01	0.9997
477.76 ± 9.13	27.22 ± 0.01	27.25 ± 0.01	0.9993
672.30 ± 11.17	32.77 ± 1.05	33.33 ± 0.07	0.9980

**Table 4:** Equations, parameters and error function values for the tested adsorption isotherm models.

Model ^1,2^	Equation	Parameters ^3^	RMSD	HYBRID	R^2^_adjsuted_
Langmuir ^1^	$q=\frac{q_{max}K_{L}C_{eq}}{1+K_{L}C_{eq}}$	q_max_ = 35.088 K_L_ = 11.760	2.777	56.052	0.956
Freundlich ^1^	$q=K_{F}{C_{eq}}^{\frac{1}{n}}$	K_F_ = 7.900 n = 4.762	1.719	18.135	0.886
Dubinin–Radushkevich ^1^	$q=q_{s}exp(-k_{ad}\varepsilon^{2})$	q_s_ = 3.10^−4^ k_ad_ = 27.719	3.079	53.815	0.674
Temkin ^1^	$q=\frac{RT}{b_{T}}ln(A_{T}C_{eq})$	b_T_ = 472.270 A_T_ = 0.530	1.861	21.275	0.852
Hill ^2^	$q=\frac{q_{sH}{C_{e}}^{n_{H}}}{k_{D}+{C_{e}}^{n_{H}}}$	q_sH_ = 7191.3 k_D_ = 744.214 n_H_ = 0.168	2.268	41.914	0.865
Redlich–Peterson ^2^	$q=\frac{K_{R}C_{e}}{1+a_{R}C_{e}^{g}}$	K_R_ = 3673.7 a_R_ = 464.983 g = 0.790	1.720	27.914	0.988
Toth ^2^	$q=\frac{K_{T}C_{eq}}{{\left(a_{T}+C_{eq}\right)}^{\frac{1}{t}}}$	K_T_ = 7.492 a_T_ = 0 t = 1.266	1.707	27.388	0.848
Radke–Prausnitz ^2^	$q=\frac{a_{RP}K_{RP}C_{eq}^{\beta_{R}}}{a_{RP}+K_{RP}C_{eq}^{\beta_{R}-1}}$	a_RP_ = 3112.800 K_RP_ = 7.493 ᵝ_R_ = 0.220	1.708	27.504	0.857

^1^ Two-parameter adsorption isotherm models. ^2^ Three-parameter adsorption isotherm models. ^3^ Units of parameters for the isotherms, whit the calculus performed with C_eq_ values expressed in g/L: Langmuir—q_max_ (mg/g) maximum monolayer coverage, K_L_ (L/g) adsorption equilibrium constant; Freundlich—K_F_ (mg/g) approximate indicator of adsorption capacity, n (dimensionless), with 1/n indicating adsorption strength, a model coefficient; Dubinin–Radushkevich—q_S_ (mg/g) theoretical saturation capacity, k_ad_ (mole^2^/KJ^2^) a measure of adsorption equilibrium constant, ε (dimensionless) Dubinin–Radushkevich isotherm constant, a model coefficient; Temkin—A_T_ (mg/g) binding constant, b_T_ (dimensionless) Temkin isotherm constant; Hill—q_sH_ (mg/g) Hill saturation capacity, k_D_ (L/g) Hill–Deboer isotherm constant, n_H_ (dimensionless) model coefficient; Redlich–Peterson—K_R_ (L/g) Redlich–Peterson isotherm constant, a_R_ (g/mg) a measure of maximum adsorption capacity, g (dimensionless) model exponent with values between 0 and 1; Toth—K_T_ (L/g) Toth isotherm constant, a_T_ (mg/g) maximum adsorption capacity, t (dimensionless) heterogeneity factor; Radke–Prausnitz—a_RP_ (mg/g) maximum adsorption capacity, K_RP_ (L/g) Radke–Prausnitz isotherm constant, β_R_ (dimensionless) model exponent.

## Data Availability

The data presented in this study are available upon request from the corresponding author.
